# Speed of Sound Measurements in Dense Siloxane D_6_ Vapor at Temperatures up to 645 K by Means of a New Prismatic Acoustic Resonator

**DOI:** 10.1021/acs.jced.2c00725

**Published:** 2023-02-03

**Authors:** Bertrand Mercier, Nitish B. Chandrasekaran, Piero Colonna

**Affiliations:** Propulsion and Power, Aerospace Engineering, Delft University of Technology, 2629 HS, Delft, The Netherlands

## Abstract

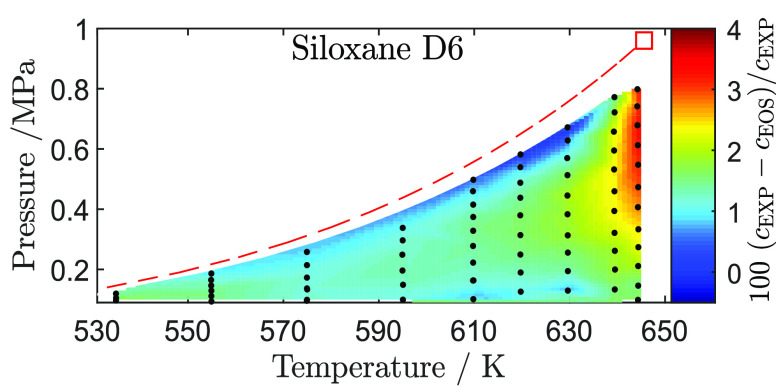

Estimating the speed of sound for the dense vapor phase of D_6_ (dodecamethylcyclohexasiloxane, C_12_H_36_O_6_Si_6_) is particularly relevant to the study of nonideal compressible fluid dynamics (NICFD), the gas dynamics of fluids whose properties depart significantly from those related by the ideal gas model. If molecular complexity is sufficiently large, dense vapor flows may exhibit so-called nonclassical gasdynamic effects, and D_6_ is a candidate for experimental studies aimed at proving for the first time the existence of these exotic phenomena. More in general, speed of sound measurements in the dense vapor phase are important for NICFD applications: for example, complex organic compounds are employed as working fluids in organic Rankine cycle power plants and the correct prediction of the dense-vapor speed of sound is of paramount importance for the design of the supersonic turbines equipping these systems. This type of measurements is challenging and thus rare, especially in the case of complex organic molecules, given the high temperature at which they must be performed, close to the temperature at which the molecule thermally decomposes. Therefore, a new prismatic resonator (the OVAR, organic vapor acoustic resonator) has been conceived, designed, and realized. It is suitable for speed of sound measurements in the vapor phase of organic compounds at temperatures up to approximately 670 K and pressures up to about 1.5 MPa. The speed of sound of D_6_ (97.4% pure) has been measured along eight isotherms between 555 and 645 K and from nearly saturated density to densities close to ideal gas conditions (compressibility factor *Z* ≈ 0.95). The estimated relative uncertainty of these sound speed measurements is 0.14%. In addition, corresponding density values have been obtained with an estimated uncertainty between 0.2 kg·m^–3^ and 1.2 kg·m^–3^.

## 1 Introduction

Fluids formed by linear and cyclic siloxane molecules, or more precisely polydimethylsiloxane polymers, are widely employed as feedstock for the production of various types of silicone polymers, in the cosmetic industry, as solvents, and (mixed) as high-temperature heat transfer fluids. The most volatile among these compounds are also used as working fluids in high-temperature organic Rankine cycle (ORC) power plants^1,2^ and recently in fundamental gas dynamics studies on nonideal compressible fluid dynamics (NICFD); see, e.g., the introductory chapter of the Ph.D. dissertation of Head^3^ for an extensive review of the literature. Siloxanes are particularly suitable for these applications and for these scientific studies because (i) their thermodynamic properties match the requirements in terms of thermodynamic cycle and of related equipment, (ii) they are thermally stable up to very high temperature (even in excess of 620 K in contact with stainless steel, if properly handled),^4−8^ (iii) they can be used as lubricants in the liquid phase, (iv) their flammability is low (e.g., compared to hydrocarbons), and (v) their toxicity does not require special precautions; see, e.g., the safety data sheet of D_6_.^9^

Among cyclic siloxanes, D_6_ (dodecamethylcyclohexasiloxane, C_12_H_36_O_6_Si_6_) might be used as working fluid of highly miniaturized high-temperature ORC power plants.^10^ Possibly more importantly, in the realm of scientific studies, D_6_ is also particularly relevant in the niche field of nonclassical gasdynamics.^11^ This branch of fluid mechanics is concerned with the study of compressible flows of dense vapors of fluids formed by complex organic molecules. For such fluids the theory predicts that classical nonlinear acoustic phenomena are inverted: for example rarefaction shock waves and compression fans are possible, as opposed to compression shockwaves and expansion fans.^12^ Gasdynamics theory prescribes that for nonclassical phenomena to occur a necessary condition is that the so-called fundamental derivative of gasdynamics
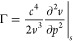
1is negative for some of the thermodynamic states that the fluid experiences throughout the flow; see, e.g., the studies of Zamfirescu et al.,^13^ Guardone et al.,^14^ Nannan et al.,^15^ and Chandrasekaran et al.^16^ Here, *c* is the sound speed, *v* is the specific volume, and *p* is the pressure. If there exist thermodynamic states in the dense vapor phase for which Γ < 0, such fluid is termed a BZT fluid (from the initials of the three scientists who first argued about the possibility of nonclassical gas dynamic phenomena, namely Bethe, Zel’dovich, and Thompson) .^17−19^ The proof for the existence of nonclassical gasdynamics has been pursued by several researchers, so far with inconclusive results.^20,21^

The Propulsion and Power group of the Delft University of Technology has researched NICFD and nonclassical gasdynamics for many years. In particular, D_6_ was chosen as the working fluid for the realization of a Ludwieg-tube-type experimental setup to generate and measure the nonclassical propagation of waves in dense vapor of organic molecules.^22,23^ Also in this case, results were inconclusive and it can be argued that one of the main difficulties is the insufficient accuracy of the available thermodynamic models, which precludes the confident identification of experimental conditions leading to the generation of the wanted phenomena.

Compared to that for other organic fluids, very few measurements of thermodynamic properties are documented in the literature for siloxanes. Moreover, the more complex the siloxane molecule is, the fewer the measurements that are available. More in general, it can be argued that the more complex an organic molecule is, the more challenging it is to perform thermodynamic properties measurements to characterize the departure of properties from those of the ideal gas, given that they have to be performed at high temperature and close to the thermal stability threshold. As a result, the current thermodynamic models for D_6_ are known to be comparatively inaccurate, and especially for thermodynamic states at high reduced temperature and pressure and close to saturation, that is, the nonideal thermodynamic states which make nonclassical gasdynamics possible.

Technical equations of state based on the Helmholtz energy formulation were initially developed for several linear and cyclic siloxanes, namely MM, MD_*x*_M with *x* = 1···5, D_4_, D_5_, and D_6_.^24−26^ The accuracy of these equations of state is however limited if compared to those available for other classes of substances because of the scarcity of experimental data sets. Information about improvements of these multiparameter equations of state models based on additional highly accurate speed of sound measurements in the liquid phase was recently published.^27−29^ However, these models were developed for all the mentioned siloxanes with the exception of D_6_.

This study is therefore motivated by the need for improving thermodynamic models for D_6_ in general, and speed of sound predictions of dense-vapor thermodynamics states in particular, in order to support experimental studies of nonclassical gasdynamics.

The speed of sound in fluids can currently be measured by means of three main techniques, with different outcomes in terms of accuracy and range of applications. The most direct method is based on the pulse-echo technique, which consists of measuring the duration of the travel of an acoustic wave through the medium over a known distance.^30,31^ This technique allows reaching an accuracy of the order of 0.01% for liquids but is not suited for vapors because their acoustic impedance is insufficient. Alternatively, the sound speed can be indirectly obtained from the modal behavior of acoustic waves in a cavity. The speed of sound is computed from the measurement of the acoustic resonance frequency of the fluid in a resonator of known dimensions. The resonator can be cylindrical^32−34^ or spherical.^35−37^ Spherical resonators allow to achieve lower relative uncertainties, typically of the order of 0.01%, but values as low as ≈1 ppm can be achieved.^36^ A comprehensive treatment of the theory of speed of sound measurements is provided by Meier.^30^ Finally, the Brillouin scattering affecting the frequency of the light dispersed by molecules can also be used to correlate it with the local speed of sound through a fluid.^38−40^ The use of this method is rare, and the associated relative uncertainty is of the order of 1%. However, it is worth noting that it features the unique advantage that no energy is transferred to the fluid during the measurement. This characteristic can be very attractive, for example, if measurements are needed in extreme temperature conditions, or in thermodynamic conditions very close to the critical point. To the knowledge of the authors, the only published sound speed measurements data related to the dense vapor of complex organic compounds are reported by Timrot et al.^41^ The scarcity of this type of data is most likely due to the difficulty of performing sound speed measurements at high temperature, because of thermodynamic state control and sealing difficulties, instrument and actuator compatibility, and also because these states are close to the thermal stability threshold of the molecule.

The authors did not find any evidence of devices which can be used to measure the sound speed at the conditions of interest for nonclassical gasdynamic studies, namely at temperatures up to 645 K and for a speed of sound value that can be as low as 50 m/s. A new apparatus had therefore to be designed, manufactured, and commissioned: the OVAR (Organic Vapor Acoustic Resonator). The OVAR is a prismatic resonator with a square cross section. Despite it being known that this type of resonator cannot provide the highest level of accuracy, it offers many advantages if the needs of the study documented here are considered. The main advantage is that, for a given characteristic dimension, it is simple to manufacture in comparison to a spherical resonator. A second advantage of the prismatic resonator is the possibility to use a larger acoustic excitation system which is required to generate a reasonable acoustic level in vapors. A square cross section for the acoustic cavity was chosen because according to the initial design an optical access should have equipped the device in order to measure also the density with an optical method based on Rayleigh Scattering.^42^

Technical details of the resonator are presented first, followed by a description of the measurement procedure. In addition to speed of sound measurements, also the density at the experimental conditions have been evaluated. An estimation of the uncertainties of both sound speed and density measurements is provided. Results are then compared with predictions of the iPRSV cubic equation of state model for D_6_. A summary of concluding remarks completes this contribution.

## 2 The Acoustic Resonator

The use of acoustic resonators for speed of sound measurements relies on the amplification of an acoustic signal inside a cavity filled with a fluid if the signal frequency matches a natural frequency of the cavity. For the OVAR, whose cavity is a rectangular cuboid of length *l*_0_ and square section of length *h*_0_ (see Figure 2), the resonance frequencies are given as a function of the speed of sound *c* by
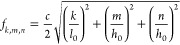
2where *k*, *m*, and *n* are integers corresponding respectively to the mode numbers in the longitudinal and the two transversal directions. If *l*_0_ is significantly larger than *h*_0_, this relation can be simplified as
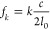
3for values of *k* such that the frequency of the longitudinal mode is significantly smaller than that of the first transverse modes. The length to width ratio of the OVAR cavity is 7.1. Equation 3 is therefore valid for *k* ≤ 6. The frequency *f*_7,0,0_ is indeed only 14% lower than *f*_0,1,0_, which means that both modes may overlap.

### 2.1 General layout

**Figure 1:**
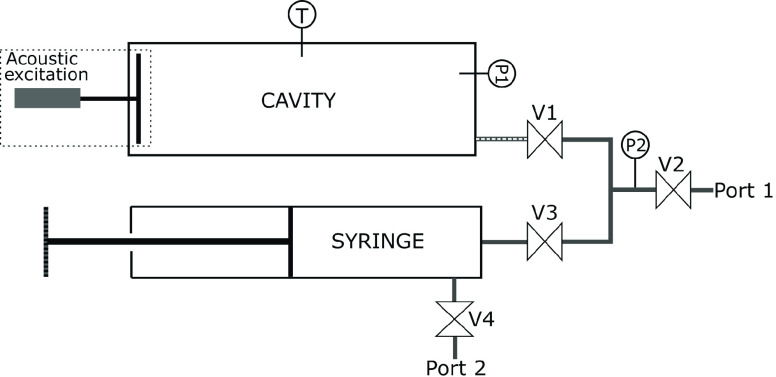
General layout of the OVAR setup.

The OVAR is made of two main components as shown in Figure 1, the cavity and the syringe. The syringe is used to inject a known volume of fluid into the cavity. In addition, it is equipped with four valves to isolate zones that need to be evacuated via Port 1 or Port 2. Figure 1 also depicts the three main sensors of the setup, namely a K-Type thermocouple sensor T that measures the temperature inside the resonator near the center of the cavity, and two pressures sensors P1 and P2 to perform fluctuation and accurate time-averaged measurements.

#### 2.1.1 Cavity

**Figure 2:**
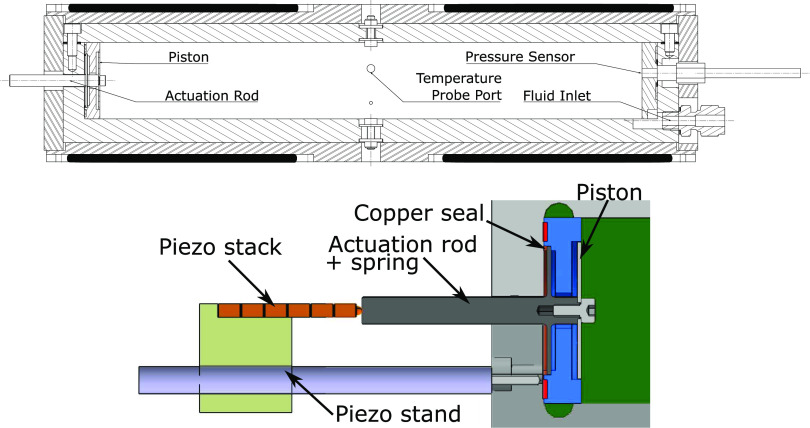
Longitudinal cross-section of the resonator showing the stainless steel cavity, the aluminum shell, and the heating elements (in black). Below, more detailed cross-sectional view of the excitation system.

The cavity (Figure 2) is milled out of a stainless steel 316L block. It is closed on the top by a stainless steel plate and terminated at both ends by two end-plates ensuring that there are sharp corners on each edge, so that the cavity shape resembles as much as possible that of a perfect cuboid. The cavity may also be equipped with optical accesses, but the openings were sealed for the purpose of the first experimental campaign, as indicated in Figure 2. The measured length of the cavity is *l*_0_ = 284 mm ± 0.2 mm, and its cross section is a 40 ± 0.05 mm square. The volume of the cavity at 293 K is 454 cm^3^ ± 1.5 cm^3^, which leads to a total volume of 461 cm^3^ ± 1.5 cm^3^ including the dead volumes behind the piston and at the corners of the cavity. This volume increases with temperature due to the thermal expansion coefficient α = (1.7 × 10^–5^ ± 3%) K^–1^, from ambient to the maximum operating temperature. The effect of the pressure onto the volume is however negligible because, for a pressure of 1 MPa, the strain is 3 × 10^–3^% in the transverse directions, and 4 × 10^–4^% in the longitudinal direction.

#### 2.1.2 Syringe

The fluid is injected into the cavity with the help of a hand-pump, namely a syringe. It is driven by rotating a handle controlling a screw of pitch 1.5 mm ± 0.0003 mm. The inside diameter of the cylinder is 30 mm ± 0.05 mm. The displacement is therefore 1.060 cm^3^ ± 0.003 cm^3^ per turn. The total volume of the syringe is approximately 100 cm^3^.

### 2.2 Instrumentation

The instrumentation consists of three sensors. The pressure is measured by a DRUCK UNIK 5000 ref X5072-TB-A2-CA-H1-PA silicon sensor (P2 in Figure 1) rated for a maximum pressure of 1.6 MPa with an uncertainty of 0.1% of the full scale, i.e., 1.6 kPa, with respect to the best fit line. After calibration it appeared that the actual uncertainty of the sensor is the addition of a 0.4 kPa bias and a relative 0.1% random error considering a day to day calibration of the offset based on atmospheric pressure. This sensor cannot withstand high temperature, thus it is mounted on the pipe between the syringe and the resonator. The 4–20 mA output is converted into voltage by a precision 250 Ω resistor, and digitized by a NI 9215 A/D converter.

The second pressure sensor is a Kulite XTEH-10L-190SM-300PSI-A (2.1 MPa maximum pressure). It is a high frequency and high temperature pressure sensor flush mounted to the inside of the cavity (P1 in Figure 1) to measure the acoustic pressure. The output is a 5–100 mV signal amplified by a custom +40 dB amplifier and also acquired with the NI 9215 module. The accuracy of this sensor is poor due to a significant drift of the calibration constant with the temperature. Therefore, it cannot be used to measure the absolute pressure inside the cavity, but it is suitable to provide the shape of the resonance curve which does not need to be converted into pressure units to determine the resonance frequency.

The temperature is measured by a calibrated K-type thermocouple mounted on a custom probe that fits in the M5 thread referred to as Temperature Probe Port in Figure 2. The tip of the thermocouple protrudes inside the cavity to ensure that the reading corresponds to the temperature of the bulk flow, and that it is not affected by the local wall temperature. This thermocouple is connected to a NI 9210 thermometer. The thermocouple was calibrated with the Fluke 9100 dry-well calibrator whose rated uncertainty is 0.5 K.

### 2.3 Acoustic Excitation

Acoustic excitation is a challenge because of the high temperature conditions affecting the entire resonator. It was therefore decided to move the actuation system away from hot parts and to connect it to an actuation rod made of stainless steel as indicated in Figure 2. The rod is in contact on one end with the actuator, a 100 V/32.5 μm Thorlabs piezo stack (reference PK3JUP1), and on the other is connected to a disk made of stainless steel acting as a piston flush-mounted with free edges in the cavity. This piston has a thickness of 0.8 mm, a diameter of 30 mm, and a first natural frequency at approximately 3.4 kHz, which is high enough to prevent any coupling considering the frequency of interest in this resonator. The rod is attached to the cavity by means of a membrane that acts as a sealing element as well as a spring. The design of this membrane required much care because such membrane must be strong enough to withstand the pressure difference between the cavity and ambient pressure, and it must simultaneously be flexible enough to be compatible with the piezo stack in terms of forces. A thickness of 0.8 mm with a free diameter of 26 mm was found to be an acceptable combination for a maximum pressure of 1 MPa. The first natural mode of the actuation system is 2.7 kHz which is also 1 order of magnitude larger than the frequencies of interest. If driven with the Thorlabs MDT694B amplifier, the maximum achievable excitation frequency is close to 500 Hz (−3 dB). The input signal for the amplifier is provided by an ELC GF467AF function generator controlled via the RS-232 protocol.

As a side note, the authors indicate that it would be difficult to realize an excitation system capable of withstanding pressures significantly higher than 1 MPa with this approach. However, the pressure limitation could be overcome with a new technique. It consists in transferring energy to the fluid as heat instead of as work by sending modulated light to an absorbent surface according to the method proposed by Suchenek and Borowski.^43^

### 2.4 Thermal Control

The temperature homogeneity along the cavity is enhanced by a shell made of 10 mm thick plates of aluminum surrounding the stainless steel walls of the cavity. The shell is fitted with four pockets (two on the top, two on the bottom) holding four heating elements. The heating elements are mica heaters with an individual nominal power of 400 W at 240 V. They are supplied with a 93 V DC tension providing a total heating power of 256 W. The temperature of each heater is monitored individually with a thermocouple located near the center of the heater within the aluminum plate, close to the interface with the stainless steel cavity. A PID (proportional-integral-derivative) controller implemented in a LabVIEW program, which also controls the resonator, generates a PWM (pulse-width modulation) signal with a one second period that drives MOSFET transistors via the solid state relays of the NI-9485 module. When stabilized, the temperature is controlled within ±0.05 K.

The resonator is covered by a 30 mm-thick insulation blanket that reduces heat fluxes toward the ambient air, and across the aluminum shells. Residual heat fluxes still exist from the heating elements to the two longitudinal ends of the resonator. A consequence is that the cavity wall temperature is not expected to be perfectly uniform. A finite element thermal analysis of the resonator was computed to estimate these variations in temperature. A uniform heat flux was imposed at the interface between the mica heaters and the aluminum plate. Thermal properties of the different materials were obtained from databases for the metals and from the manufacturer specifications for the insulation material. The convection coefficient between the outer surface and the atmosphere was chosen to fit the measured surface temperature. The thermal analysis put into evidence the possibility that cold spots may occur on the inner wall of the cavity. These surfaces are nearly 1.5 K colder than the area directly facing the positions of the four heaters. This phenomenon may become significant if the thermodynamic state for which the speed of sound measurement is planned is close to the dew point because local condensation may occur. Sufficiently far from conditions of local condensation, the homogeneity of the fluid can be achieved if the heat transfer through the fluid is largely due to natural convection and conduction is minimal. Such condition of convection dominance can be verified by means of the Rayleigh number *Ra* defined as
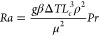
4where *g* is the acceleration due to gravity, β, ρ, μ and *Pr* are the isobaric and thermal expansion, the density, the dynamic viscosity, and the Prandtl number of the fluid; Δ*T* and *L*_*c*_ are the characteristic temperature difference and characteristic length of the considered configuration. The so-called critical Rayleigh number is of the order of 10^3^, whereby higher values indicate the dominance of convection. If the fluid is siloxane D_6_, its thermodynamic properties can be estimated by means, for example, of an in-house server software implementing various thermodynamic and transport properties models for pure fluids and mixtures.^44^ In particular, the iPRSV cubic equation of state^45^ was employed to calculate thermodynamic properties, and the Chung et al. model^46^ for transport properties. The Rayleigh number was computed for various experimental conditions with *L*_*c*_ = 0.04 m and Δ*T* = 1 K and, as expected, its value is lower for low values of pressure *p* and high temperature *T* because in such thermodynamic states density is lower and viscosity higher. For *p* = 100 kPa and *T* = 650 K, *Ra* = 5 × 10^5^, thus a much larger value if compared to the critical Rayleigh number. Natural convection is therefore the dominant heat transfer mechanism within the fluid, indicating that the fluid temperature can be considered as homogeneous, and the thermal boundary layer can be neglected. *Ra* becomes as large as 10^8^ for *p* = 0.8 MPa and *T* = 650 K.

It can be concluded that, if the working fluid is siloxane D_6_, or other similar siloxanes, the temperature measured by the thermocouple protruding inside the cavity can be considered as an accurate value contributing to the identification of the thermodynamic state of the fluid inside the cavity. This temperature is however always slightly lower than the setpoint temperature of the heaters due to cold spots occurring on surfaces not subjected to active heating.

## 3 Experimental Method

### 3.1 Fluid Characteristics and Purification

Before the D_6_ sound speed measurement campaign, siloxanes D_4_ and D_5_ were utilized to calibrate the measurement system, given that high accuracy speed of sound experimental data at moderate temperatures in the vapor phase of these fluid are reported.^47^ Both fluids were produced by Tokyo Chemical Industry and certified to be 99.9% pure. The experimental campaign aimed at obtaining dense vapor sound speed values for siloxane D_6_ was based on fluid samples obtained from Dow Corning. The purification level that can be obtained for D_6_ is lower and equal to 97.4%. According to the supplied gas chromatography analysis, the samples also contain 1.7% of D_5_, 0.5% of D_7_, and traces of lighter siloxanes and water. The main properties of these siloxanes are summarized in Table 1, and main information about the D_6_ sample is provided in Table 2.

**Table 1:** Main Properties of the Three Cyclic Siloxanes Involved in the Measurement Campaign^24,25^

	D_4_	D_5_	D_6_
CASRN	556-67-2	541-02-6	540-97-6
Boiling temperature at 101.3 kPa (K)	448	484	518
Molecular Mass *M*(g/mol)	296.6	370.1	444.9
Density ρ at 101.3 kPa, 298 K (kg/m^3^)	950	954	963
Critical pressure *P*_c_ (MPa)	1.33	1.16	0.961
Critical temperature *T*_c_ (K)	586	619	646
Critical density ρ_c_(kg/m^3^)	307	293	279

**Table 2:** Composition of the D_6_ Sample According to the Supplier’s Analysis

Material	CASRN	Molar mass/g· mol^–1^	Mole fraction /%
D_6_	540-97-6	444.92	97.374
D_5_	541-02-6	370.77	1.666
D_7_	107-50-6	519.07	0.458
D_3_	541-05-9	222.46	0.066
D_4_	556-67-2	296.64	0.039

Dehydration, thus extraction of dissolved water molecules, is mandatory because water can adversely affect the thermal stability of organic fluids.^7^ All the three fluid samples were dehydrated by keeping beads of 3 Å molecular sieves immersed into the samples for several days.

Gases dissolved in the fluid are also detrimental with respect to the thermal stability of the fluid molecules and the accuracy of speed of sound measurements. To mitigate this issue, vacuum degassing of the fluid samples was adopted, namely the fluid samples are left under vacuum in a large vessel for at least 12 h and then the vacuum pump is operated to extract the incondensible substances from the vessel. This process was repeated every time a fluid sample was brought to high temperature because it was found that a detectable amount of incondensible gases are produced, most likely due to the thermal decomposition of siloxanes when kept at high temperature for a long period of time. If light gases are not extracted, they cause a day to day increase of the measured speed of sound because they are released during the next heating. Other possible products of thermal decomposition which were also observed were visible black particles, possibly black carbon conglomerates forming after several hours of exposure of the fluid to high temperature. No quantitative measurements of the concentration of these particles were carried out, but it seemed that they only appeared when approaching temperatures of 630 K and above, without significantly affecting the value of the measured speed of sound. The thermal stability threshold of cyclic siloxanes in stainless steel of approximately 630 K is in agreement with previous findings.^5^

### 3.2 Measurement Procedure

Before starting any experiment, the cavity is connected to the vacuum pump through Port 1, valves V2 and V1 are open, and the pump is actuated (see Figure 1). V2 is then closed, and the heating system is turned on to reach the desired temperature set point. The pressure rises slightly with the temperature because residual siloxanes from the previous experiment evaporate; the vacuum pump is therefore used also to extract these residual vapors. At the same time, the syringe is filled with the desired quantity of purified fluid via port 2. The pressure of the syringe is then set to vacuum conditions by connecting the vacuum pump to Port 2.

When the temperature has reached the set point, valve V1 is closed, and V3 is opened. The syringe is again connected to the vacuum pump through Port 1, and vacuum is set. V2 is closed, and the fluid is pushed with the syringe to fill the pipes. Valve V1 is then opened and the desired amount of fluid can be injected by rotating the syringe handle with the desired angle. A temperature drop of the fluid inside the cavity is instantaneously observed. Speed of sound measurements can only start after the temperature has stabilized again and reached the setpoint. The measurements are repeated at least three times for each configuration, which together with constant temperature and pressure, ensures that steady state is reached.

Siloxane D_4_, D_5_, and D_6_ were all tested in the resonator, therefore care was taken to avoid fluid contamination. To this end, if the tested fluid is the same as the one used for the previous measurement, the syringe is used to push more fluid into the resonator in order to reach a new thermodynamic state. If the fluid previously used was different, then the entire system is flushed and the procedure is repeated with fresh fluid. This cleaning procedure is repeated until the speed of sound remains constant after the fluid is replaced. This condition indicates that there is no significant traces of contaminants and is generally reached after three to four iterations.

A further precaution during the operation of the resonator concerns the fluid injection. The mass of injected fluid is a function of the fluid density inside the syringe, and hence of the fluid temperature. Though the outer surface temperature of the syringe is monitored, it is critical to never transfer hot fluid from the cavity to the syringe to avoid any rapid increase in temperature of the fluid stored inside the syringe which can not be captured by the temperature sensor. Successive speed of sound measurements were therefore always conducted by increasing the fluid density in the cavity. If, for any reason, fluid needed to be extracted, a long delay was imposed to ensure that the temperature inside the syringe had reached ambient temperature.

### 3.3 Estimation of the Speed of Sound

**Figure 3:**
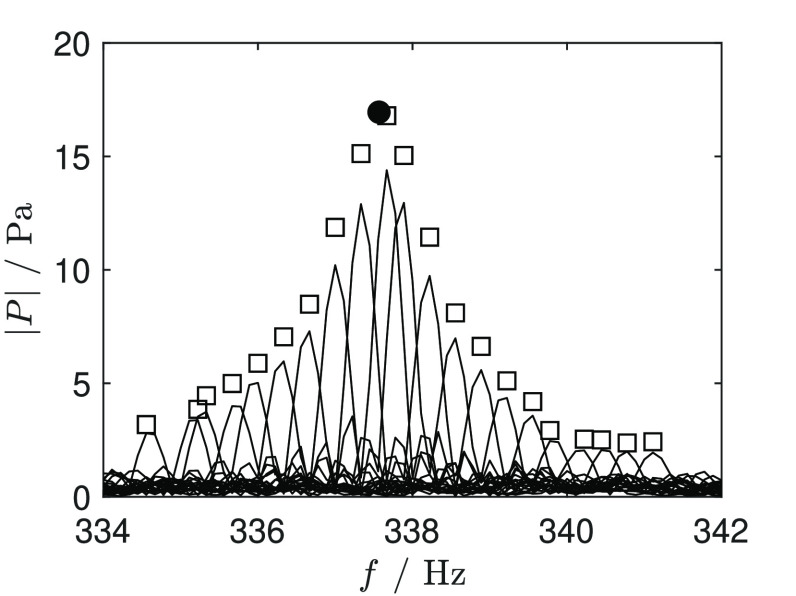
Fourier transforms of the recordings for the estimation of the resonance frequency in D_6_ at a 255 kPa and 629.8 K: □, maxima of each individual Fourier transform (amplitude is divided by 3); •, maximum of the resonance peak (amplitude divided by 3).

The speed of sound is determined from the frequency of the acoustic resonance of the cavity filled with the fluid using eq 3 with the cavity length corrected for thermal expansion. The frequency response of the cavity was scanned by sending a sinusoidal signal to the piezoelectric actuator and by measuring the acoustic response with the Kulite pressure sensor. The range of emitted frequencies was chosen to encompass the entire resonance peak of the second longitudinal mode, typically over a range of 5 to 7 Hz and with steps of *df* = 0.33 Hz. The excitation is started approximately 0.5 s before the beginning of the recording to provide enough time for the resonance to reach its maximum amplitude. The recording time is 1/*df* = 3 s for consistency with the next postprocessing step. Once the scanning is done, the resonance of the cavity is described by a collection of records as shown in Figure 3.

It was observed that the actuation frequency differs slightly from that prescribed to the signal generator because of a bias due to controlling this device with serial communication. The next step is therefore to identify more precisely the excitation frequencies, and the amplitude of the pressure signal *p*(*t*) at each excitation frequency. Frequency identification was performed in the frequency domain using the Fast Fourier Transform *P*(*f*) of the signal *p*(*t*). The frequency resolution of the Fourier transform was improved by a factor of 3 by means of so-called zero-padding, thus *df*/3 = 0.11 Hz. The obtained resolution could be further improved by windowing the pressure signal with a Gaussian weighting and by fitting the peak in the FFT with a Gaussian function as explained by Gasior and Gonzalez.^48^ Yet 0.11 Hz was found to be sufficient for the purpose of this study. The rms value  of the pressure signal at the excitation frequency is obtained following Parseval’s theorem as
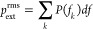
5where *f*_*k*_ is the *k*^th^ frequency which is an index identifying the range of the peak corresponding to the excitation frequency in *P*(*f*). Figure 3 shows an exemplary result obtained by applying this method to the recording of one scan of the cavity resonance.

The final step consists in determining the resonance frequency of the cavity from the individual records. A common practice is to fit the envelope of the resonance peak formed by the previously determined points with a Lorentzian curve. However, a more robust, time-effective and sufficiently accurate method was devised, given the relatively large uncertainty of the measurement system. The resonance frequency is determined by averaging the peak frequencies within the corresponding record, weighted by their amplitudes. Only the records with an amplitude of at least half of the maximum amplitude were considered. The result is a barycentric frequency close to the frequency at which the maximum acoustic pressure would have been measured (this point is shown by a black dot in Figure 3).

The obtained frequency is not exactly the resonance frequency because it is influenced by all the sources of energy dissipation, see the detailed study of Liu et al.^34^ A better estimation of the resonance frequency is generally obtained by adding the half width at half-maximum (HWHM) of the resonance peak to the central frequency of the peak. However, the observed HWHM value is at least an order of magnitude larger than that expected from the source of errors summarized by Liu et al.^34^ and this can be explained as follows.

- Errors may arise from interactions between the fluid and the cavity shell. Zhang et al.^49^ proposed a model to estimate the effect of this interaction for a resonator with a circular cross-section. This model is applied to the OVAR by approximating the cross-section of the OVAR to be circular with the same cross-sectional area as the actual square prism. This results in a perturbation of the peak frequency of the order of 0.01%. This perturbation is small because of the large difference between the speed of sound in D_6_ vapor and in stainless steel.
- Frequency shifts can be introduced by the interaction between the acoustic modes and the vibrational relaxation of the molecule; however, this is not relevant in the frequency range that is considered in this resonator.
- Perturbation due to the wave propagation in the inlet duct is also expected to be weak because the duct diameter is only 0.8 mm and is filled with liquid D6, which implies the existence of a reflective boundary at the liquid/vapor interface.
- Thermoviscous dissipation also affects the frequency response of the cavity. Its effect can also be estimated following Zhang et al.^49^ However, since the correction reported by Zhang et al. is for a cavity of circular cross-section, it is calculated also in this case for an equivalent cavity of diameter *a* = 51 mm with perimeter equal to that of the 40 mm × 40 mm square cavity. For longitudinal modes, the viscous perturbation Δ*f*_*v*_/*f*_0_ and the thermal perturbation Δ*f*_*T*_/*f*_0_ are
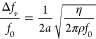
6and
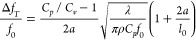
7where η is the dynamic viscosity, λ is the thermal conductivity, and *C*_*p*_ and *C*_*v*_ are the specific heat at constant-pressure and constant-volume, respectively. These properties for D_6_ have been estimated, albeit with fairly large uncertainty, by means of an in-house program^44^ implementing a Helmholtz multiparameter equation of state model^25^ (*C*_*p*_ and *C*_*v*_) and the Chung et al. model^46^ (η and λ). Noting that the relative uncertainty associated with these property values cannot be easily quantified, Δ*f*_*v*_/*f*_0_ = 1.4 × 10^–4^, Δ*f*_*T*_/*f*_0_ = 1.8 × 10^–4^ at *T* = 630 K and *p* = 255 kPa, with *C*_*p*_ = 1992 J/kg·K, *C*_*v*_ = 1957 J/kg·K, η = 1.1 × 10^–5^ Pa·s, λ = 3 × 10^–2^ W/m·K, *f*_0_ = 338 Hz.

The dominant perturbation therefore arises from the viscous and thermal boundary layers. Two more sources of dissipation specific to this resonator could not be evaluated, namely, the viscous dissipation occurring at the gap between the piston and the cavity wall, and the dissipation due to phase change at the interface between vapor and liquid in the inlet pipe. It is however unlikely that their effect is 2 orders of magnitude larger than that of thermoviscous dissipation at the walls. All added together, the broadening of the full width at half-maximum (FWHM) due to wave dissipation mechanisms should not exceed 0.1 Hz. As a consequence, the nearly 2 Hz FWHM that can be observed in Figure 3 must be the result of another perturbation. It is conjectured that this somewhat large FWHM is due to albeit small variations of temperature within the cavity. This statement is supported by the observation that the FWHM tends to decrease if the resonator is kept at rest for a longer period of time, or if it is operated at a higher density, which causes better mixing of the fluid due to buoyancy. These variations introduce disturbances in local sound speed and in local flow velocity in the cavity due to natural convection. However, because the cavity is closed, the average velocity in any cross-section of the cavity must be zero following the continuity constraint. Also, the temperature fluctuations are small enough to assume that their effect on sound speed is linear, thus the bulk sound speed within the resonator is that associated with the average temperature. As a consequence, these fluctuations in temperature are not expected to cause any bias in the peak frequency of the resonance despite locally disturbing the wave fronts, causing some broadening of the spectrum. This was confirmed by the experimental observation that keeping the resonator at steady-state for a long time does not cause a sizable change of the measured central frequency of the resonance peak. Based on this remark, and considering that exposure of the fluid at high temperature increases the amount of thermal decomposition products, thus affecting the purity of the fluid sample, it can be argued that the obtained FWHM is acceptable and there is no need to wait until a further homogenization of the temperature. It is therefore assumed that the frequency of maximum pressure fluctuation coincides with the resonance frequency.

### 3.4 Calibration of the Resonator

The linear relation between the speed of sound and the resonant frequency of the cavity is described by eq 3 for a perfect cavity; however, this equation is not expected to accurately model the actual resonator whose geometry is made of three coupled cavities. The main cavity is connected to two residual cavities, one that is situated behind the piston (see Figure 2) and one at the fluid inlet. It was therefore decided to determine a correction for eq 3 by a calibration of the resonator. This process rests upon the comparison of the measured speeds of sound with reference measurements available in the literature. Despite no data being available for siloxane D_6_, accurate sound speed measurements are reported in the literature by Nannan et al.^47^ for D_4_ and D_5_ at conditions of pressure and temperature matching with the capabilities of the OVAR. The thermodynamic states selected for calibration are summarized in Table 4. A sufficient high-purity amount of both D_4_ and D_5_ from the same batch of fluid that was used for the experiments documented by Nannan et al.^47^ was still available in the laboratory and was used for calibration. Importantly, the possibility of performing this type of calibration with the same fluids allows mitigation of the influence of a possibly relevant source of error, namely the rather large amount of impurities in the D_6_ samples used for the measurement campaign. Sourcing siloxanes with a higher level of purity proved to be impossible as of the time of the documented experiments. Performing a calibration with more conventional and well-characterized fluids such as CO_2_ was considered as an alternative. However, the resonant frequencies associated with fluids made of simpler molecules at high temperature are out of the range of the excitation capability of the apparatus. In addition to the correction of systematic biases due to the geometry of the resonator, performing this calibration also allows the identification of random variations in the sound speed measurements, and therefore makes it possible to determine an uncertainty base for the statistics of the observed errors.

The calibration was performed in two stages. A first set of measurements using D_5_ and D_4_ as working fluids was performed before the D_6_ experimental campaign. A second set of calibration measurements with D_4_ was executed following all the D_6_ sound speed measurements in order to asses a possible time-drift of the resonator properties. For each of the thermodynamic states listed in Table 4, the speed of sound *c* measured with the OVAR was corrected for the small difference in pressure Δ*p* and in temperature Δ*T* with respect to the reference values with
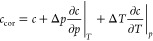
8where the partial derivatives of the speed of sound are computed using an in-house program^44^ implementing the multiparameter equation of state model of D_6_ documented by Colonna et al.^25^ Deviations between the temperatures and pressures determining the thermodynamic state of the fluid in the cavity and the reference values were within ±0.1 K and ±0.05 kPa. The inaccuracy of the thermodynamic model is not expected to play any significant role in the calibration process given the small magnitude of these corrections.

**Figure 4:**
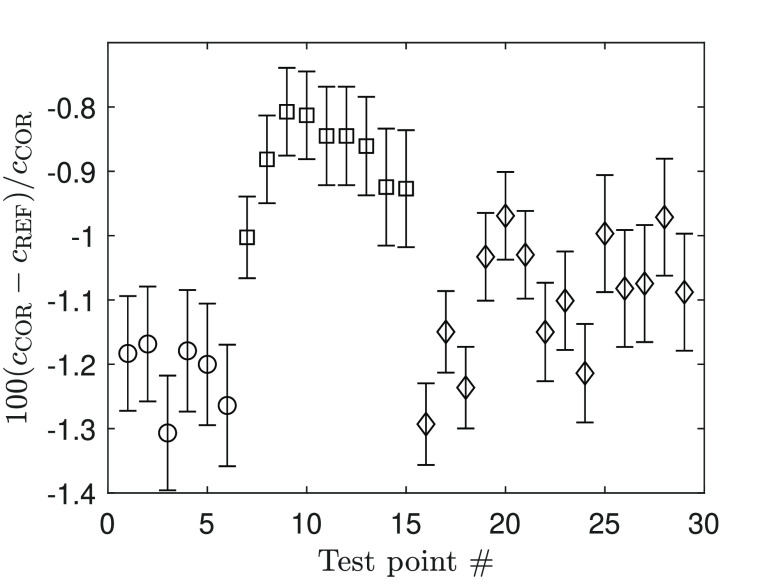
Deviation between measured sound speed values and reference values of Nannan et al.^47^ Error bars show 1σ uncertainty derived from pressure and temperature uncertainties: ○, D_5_; □, D_4_ before D_6_ test campaign; ◇, D_4_ after D_6_ test campaign.

The same partial derivatives of the speed of sound are also used to estimate the effect of the pressure uncertainty *u*(*p*) and of the temperature uncertainty *u*(*T*) discussed in section 2.2 on the sound speed uncertainty *u*(*c*) following
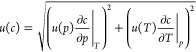
9Deviations between the measured speed of sound and the reference values are listed in Table 4 and displayed in Figure 4. Deviations of approximately −1.2% observed for D_5_ before the D_6_ measurement campaign are consistent with those close to −1.1% observed for D_4_ after the D_6_ measurement campaign. However, speed of sound values measured in D_4_ before the D_6_ test campaign were approximately 0.2% higher than those measured after the test campaign, which is statistically significant. This is most probably the result of the presence of a small amount of noncondensable gases in the fluid sample, either because air leaked inward before the charging procedure or because of the imperfect degassing of the fluid sample before the measurement campaign. The sensitivity of speed of sound to the presence of light gases is large because of the much higher value of their speed of sound. The influence of these impurities is more pronounced if the thermodynamic state is at low density. For example, if a measurement in D_4_ at 140 kPa and 500 K is considered, an error of 0.2% would be caused by a small amount of air featuring a partial pressure of 0.07 kPa. This small amount of air may not be detected because of the ±0.4 kPa offset of the sensor. For this reason, test points 7 to 15 in Figure 4 were discarded as far as the computation of the calibration constant is concerned. The average error based on all other measurements is −1.12% with a standard deviation of 0.109%. A value of 0.03% is added to this standard deviation to account for the sum of the errors caused by thermoviscous dissipation and by interactions with the shell of the resonator. This value of 0.03% is nonetheless most probably overestimated because the D_4_ and D_5_ reference measurements were also affected by similar thermoviscous dissipation; therefore, these effects are at least partially accounted for by means of the calibration constant. Table 3 summarizes the breakdown of the uncertainties. The speed of sound *c* is therefore expressed in terms of the measured speed of sound *c*_EXP_ as
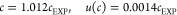
10

**Table 3:** Sources and Corresponding Values of Uncertainty Affecting the Measured Quantities

	Temp	Pressure	Density	Sound speed
Calibration	0.5 K	0.1%^a^		0.11%^a^
Bias		0.4 kPa	0.03 kg·m^–3^	
Mass injection			0.15 kg·m^–3^	
Syringe displacement			0.35%^a^	
Cavity volume			0.33%^a^	
Thermal expansion			1.5 × 10^–4^(*T* – 293)^a^	
Δ*f*_*v*_/*f*_0_				0.01%^a^
Δ*f*_*T*_/*f*_0_				0.01%^a^
Shell interaction				0.01%^a^

a Relative uncertainty.

**Table 4:** Thermodynamic States and Reference Speeds of Sound Selected for the Calibration of the OVAR. Values Are Taken from the Work of Nannan et al^47^

Fluid	*T*/K	*p*/kPa	*c*/m/s	*u*(*c*) × 10^4^/m/s
D_4_	495.00	143	109.7	283
D_4_	495.00	160	108.4	105
D_4_	495.00	225	103.4	31.8
D_5_	510.00	92.8	100.8	5.9
D_5_	510.00	103	99.89	82.5
D_5_	510.00	111	99.98	120

### 3.5 Estimation of the Density

The use of a custom-made syringe for injecting the fluid also allows evaluation of the volume and the mass of the fluid introduced into the cavity whose volume is known, and thus the density of the vapor corresponding to the set thermodynamic state in the cavity. Assuming that the pipes are initially empty, the inner volume of the pipes must be subtracted from the total volume of fluid displaced by the syringe to determine the amount of fluid introduced in the cavity. Under this assumption, the error introduced by relative changes in density during the measurement of an isotherm was measured to be 0.15 kg/m^3^ and corresponds to the uncertainty of 1/16 turn associated with the reading of the position of the handle that controls the piston in the syringe on which the estimation of the mass of liquid introduced into the cavity is based. In addition, relative uncertainties with respect to the estimation of the cavity volume arise from (i) the uncertain volume (0.33%), (ii) the thermal expansion relative uncertainty which can be evaluated as 1.5 × 10^–6^ · (*T* – 293), and (iii) the relative uncertainty related to the syringe volume displacement (0.35%).

However, a further day to day variation between repeated measurements at the same temperature and pressure of up to 1 kg/m^3^ was observed. This source of error was later identified to result from the presence of liquid D_6_ in the pipe connecting the pressure sensor to the inlet because the vacuum pump was not able to remove it completely, probably due to the effect of capillary forces.

The density estimation is therefore affected by an unknown offset which changes every time the cavity is emptied, thus every time measurements along a new isotherm are performed. This bias could be mitigated provided that the offset can be estimated. This can be done since all measurements were performed along isotherms starting from low density conditions at which the compressibility factor is larger than 0.95; hence, the departure of the thermodynamic properties from those of the ideal gas is very small and the existing equation of state models are expected to be accurate. It was therefore decided to estimate the offset as the average of the difference between the measured density and the one estimated with the iPRSV cubic equation of state^45^ for the three measurement conditions at lowest pressure along each isotherm. The error introduced by this correction cannot be accurately quantified because no density measurement is documented in the literature. An estimate of magnitude of this random error can nevertheless be given based on the accuracy of the equation of state model of siloxane D_5_, which was initially devised within the same study as that of D_6_^25^ and it was recently compared to a more accurate update of the model.^29^ Thol et al.^29^ reports a relative uncertainty of the order of 0.2% for the vapor density at low pressure. This would imply that the density estimates obtained with the thermodynamic model for D_6_ are affected by an additional error of 0.03 kg/m^3^ for the isotherm at the lowest temperature of 544 K, and the error is even less at higher temperature. This potential error is added to the uncertainty budget for density whose components are summarized in Table 3.

## 4 Measurements

### 4.1 Thermodynamic States

**Figure 5:**
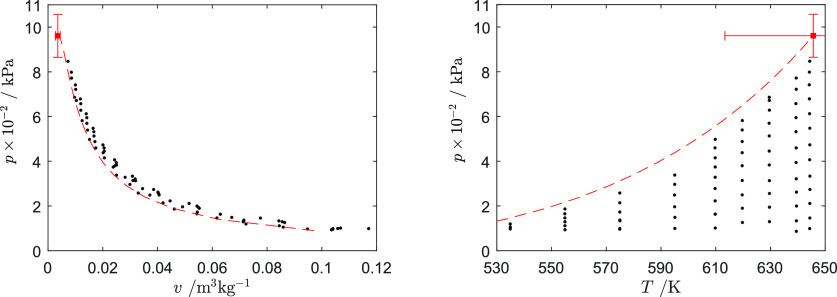
Thermodynamic states in the vapor phase (●) at which the speed of sound was measured in the *p*–*v* and *p*–*T* diagram of D_6_. The dew line computed with the iPRSV thermodynamic model (red dashed line) and the critical point estimate (red ■) together with the associated uncertainties are also indicated.

The thermodynamic states at which the speed of sound was measured are reported in Figure 5. They cover eight isotherms ranging from 544 to 644 K, hence reduced temperature *T*_r_ = *T*/*T*_c_ from *T*_r_ = 0.859 to *T*_r_ = 0.997. The pressure was varied between approximately 100 kPa up to approximately 90% of the dew pressure. All thermodynamic states feature a value of the compressibility factor *Z* = *pv*/*RT* between 0.95 and 0.44; therefore, they all feature a rather large departure from the ideal gas state. Experimental conditions and measurements are summarized in Table 5.

**Table 5:** Experimental Values of Speed of Sound *c* and Density ρ for D_6_ as a Function of Temperature *T* and Pressure *p*^a^

*T*/K	*p*/kPa	*u*(*p*)/kPa	ρ/kg·m^–3^	*u*(ρ)/kg·m^–3^	*c*/m·s^–1^
535.1	97.0	0.5	10.6	0.3	94.0
535.1	105.3	0.5	11.6	0.3	93.0
535.1	119.5	0.5	13.8	0.3	91.5
555.1	93.4	0.5	9.7	0.2	96.9
555.0	111.4	0.5	11.8	0.3	95.5
555.1	129.3	0.5	14.0	0.3	93.9
555.0	146.3	0.5	16.2	0.3	92.3
555.0	164.9	0.6	18.4	0.3	90.5
555.0	186.3	0.6	21.7	0.3	88.2
575.1	96.0	0.5	9.6	0.2	99.0
575.1	98.9	0.5	9.6	0.2	98.9
575.1	133.6	0.5	14.0	0.3	96.4
575.1	136.8	0.5	14.0	0.3	96.1
575.1	172.7	0.6	18.3	0.3	93.3
575.1	214.1	0.6	23.8	0.3	89.8
575.1	257.9	0.7	30.3	0.4	85.7
595.2	099.3	0.5	9.5	0.2	101.3
595.2	149.0	0.5	14.9	0.3	98.3
595.1	196.2	0.6	20.4	0.3	95.1
595.1	248.9	0.6	26.9	0.4	91.3
595.2	296.5	0.7	33.4	0.4	87.4
595.2	338.0	0.7	40.0	0.5	83.6
610.0	101.2	0.5	9.4	0.2	102.6
609.9	163.2	0.6	15.9	0.3	99.5
610.0	163.3	0.6	15.9	0.3	99.5
609.9	222.6	0.6	22.4	0.3	95.9
609.9	277.8	0.7	29.0	0.4	92.2
609.9	328.3	0.7	35.5	0.4	88.7
609.9	374.1	0.8	42	0.5	85.0
609.9	415.1	0.8	48.6	0.5	81.3
609.9	459.6	0.9	57.3	0.6	77.0
610.0	497.6	0.9	66.0	0.7	72.8
619.9	126.0	0.5	11.6	0.3	102.8
619.9	189.7	0.6	18.1	0.3	99.5
619.8	249.3	0.6	24.6	0.4	96.1
619.8	313.9	0.7	32.3	0.4	92.1
619.8	379.7	0.8	41.0	0.5	87.4
619.8	438.0	0.8	49.7	0.5	82.8
619.8	487.8	0.9	58.4	0.6	78.4
619.8	539.6	0.9	69.3	0.7	73.2
619.9	581.8	1.0	80.1	0.8	68.1
629.8	129.6	0.5	11.7	0.3	103.5
629.8	194.4	0.6	18.2	0.3	100.8
629.8	255.7	0.7	24.8	0.4	97.6
629.8	313.1	0.7	31.3	0.4	94.4
629.7	383.1	0.8	40.0	0.5	90.0
629.7	445.3	0.8	48.7	0.5	85.8
629.7	513.0	0.9	59.6	0.6	80.3
629.6	569.7	1.0	70.4	0.7	75.2
629.7	628.3	1.0	83.5	0.8	69.2
629.7	671.8	1.1	96.6	0.9	63.7
629.7	686.0	1.1	103.1	0.9	61.7
639.6	086.5	0.5	7.5	0.2	107.7
639.6	133.3	0.5	11.9	0.3	105.4
639.7	199.6	0.6	18.4	0.3	102.4
639.6	261.8	0.7	24.9	0.4	99.2
639.6	321.4	0.7	31.4	0.4	96.2
639.5	394.0	0.8	40.1	0.5	92.2
639.5	459.5	0.9	48.8	0.5	88.1
639.5	531.9	0.9	59.7	0.6	83.1
639.5	594.8	1.0	70.6	0.7	78.2
639.4	657.5	1.1	83.6	0.8	72.8
639.6	721.6	1.1	98.8	0.9	66.4
639.5	772.0	1.2	116.2	1.0	60.0
644.5	98.7	0.5	8.5	0.2	107.6
644.5	146.0	0.5	12.9	0.3	105.3
644.5	211.2	0.6	19.4	0.3	102.7
644.6	273.8	0.7	25.9	0.4	99.8
644.6	333.3	0.7	32.5	0.4	96.9
644.5	406.6	0.8	41.2	0.5	93.0
644.5	473.2	0.9	49.8	0.5	89.1
644.5	547.5	0.9	60.7	0.6	84.4
644.4	612.4	1.0	71.6	0.7	79.7
644.4	678.7	1.1	84.6	0.8	74.4
644.3	741.5	1.1	99.8	0.9	68.3
644.4	798.3	1.2	117.3	1.0	62.3
644.5	847.1	1.2	136.8	1.2	56.1

a Standard uncertainties *u* are *u*(*T*) = 0.5 K, and *u*(*c*) = 0.0014*c*.

### 4.2 Comparison with Sound Speed and Density Values Computed by Means of a Cubic Equation of State Model

The obtained measurements are compared with speed of sound and density values calculated with a thermodynamic model formed by the iPRSV cubic equation of state^45^
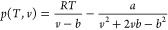
11where *v* = 1/ρ is the specific volume, and *a* and *b* are fluid-specific coefficients and by a temperature-dependent polynomial expression for the isobaric ideal-gas heat capacity.^47^ This model was chosen because the more complex multiparameter model^25^ is fitted on points generated with this cubic equation of state as far as the vapor phase is concerned, due to the lack of any experimental data in this thermodynamic region. The iPRSV model is also implemented in an in-house program for the calculation of fluid thermophysical properties^44^ which was used for all computations.

#### 4.2.1 Density

**Figure 6:**
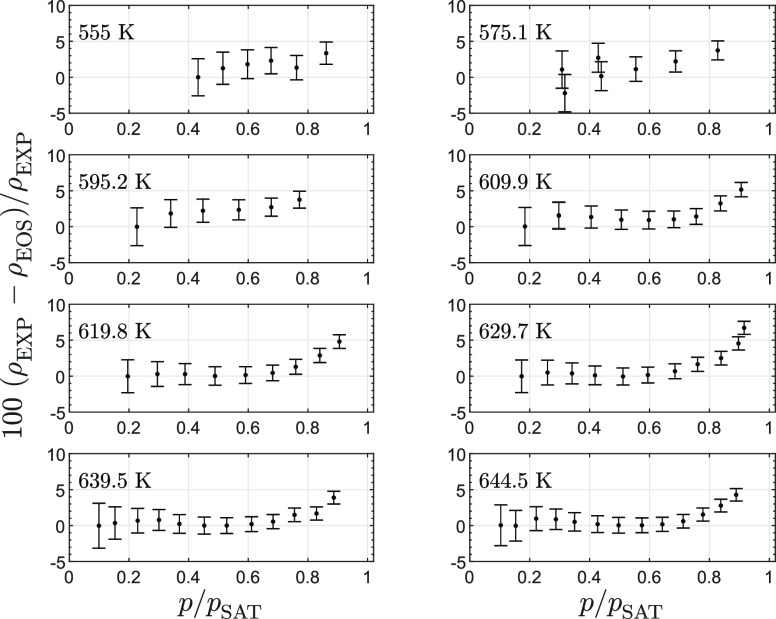
Relative deviation in density between the experimental measurements and the density estimates computed with a cubic equation of state model^44,45^ against the pressure normalized by the saturation vapor pressure at the given temperature.

Discrepancies between the density calculated with the iPRSV equation of state and density measurements corrected with the method described in section 3.5 are displayed in Figure 6. A first remark about the results reported in this figure is that, for isotherms with *T* > 595 K (*T*_r_ > 0.92), the relative difference lies within 0.0% and 1.5% for values of the normalized pressure *p*/*p*^sat^ up to approximately 60%. This fairly good agreement can be expected because it is a constraint of the density correction method. However, the fact that the difference between calculated and experimental value remains almost constant over this range of pressure has two consequential implications. First, as expected the model predicts well the variation of density with the variation of pressure at moderate *p*/*p*^sat^. Second, and consequently, the hypothesis that the equation of state provides relatively accurate estimates of the vapor density for isotherms with *T* > 595 K is valid, and the corresponding density measurements can be considered as reliable.

For isotherms with *T* ≤ 595 K, the relative difference between the measured density and that calculated with the equation of state becomes larger. This can be partially explained by the larger relative uncertainties in the measurement of the density at those conditions.

Above *p*/*p*^sat^ = 0.7, the equation of state estimate is increasingly different from the measured density: the value calculated with the equation of state is lower than the measured value by as much as 5% at *p*/*p*^sat^ = 0.9 and this difference may increase at pressures that are closer to the dew pressure.

One possible explanation for this deviation is the inaccurate estimation of the saturated vapor density, which is possibly overestimated by approximately 10% (see Figure 6). This error may be the consequence of the lack of experimental data for the vapor–liquid critical point, which have been estimated starting from the critical point values of lighter siloxanes.^25^ Colonna et al.^25^ reported uncertainties that were defined arbitrarily by the DIPPR as 10% for the pressure, 5% for the temperature, and 25% for the specific volume. These uncertainties are possibly exaggerated, but they illustrate the issues caused by the lack of experimental information for this fluid.

#### 4.2.2 Speed of Sound

**Figure 7:**
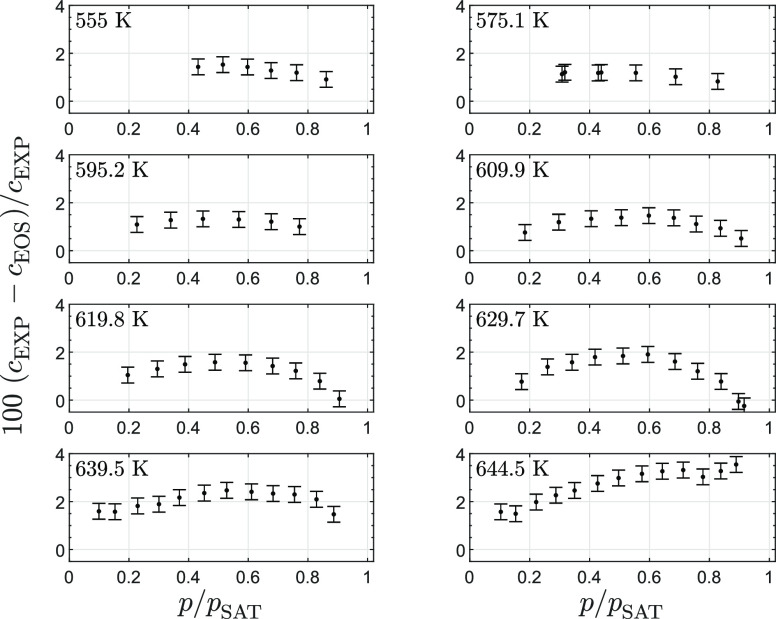
Relative deviation between speed of sound measurements along isotherms and values calculated with the iPRSV cubic equation of state^44,45^ plotted against the pressure normalized by the value of the saturation pressure at the given temperature. Error bars show the 95% confidence interval.

Differences between speed of sound measurements and values calculated with the iPRSV model^45^ are reported in Figure 7. For all isotherms, the speed of sound calculated with the model is lower by approximately 1% to 1.5% at the lowest pressure, which is a significant difference if compared to the typical uncertainty of speed of sound estimations. At those conditions, the model predicts that the ideal gas departure implies a reduction of the speed of sound from ideal gas values of 4% to 6% over the range of tested temperatures. Experimental measurements therefore show that the thermodynamic model overestimates the ideal gas departure, even in the dilute vapor region. This deviation may be the result of the large uncertainty affecting the estimation of the ideal gas heat capacity of D_6_ used as an input for the thermodynamic model (data were taken from the work of Nannan et al.^47^). It could also be argued that impurities in the fluid impact speed of sound measurements. However, the D_5_ and D_7_ traces in the tested fluid sample are estimated to alter the ideal gas speed of sound by just 0.1%.

Figure 7 shows that the discrepancy between measured and calculated values remains within the same order of magnitude for all isotherms, with a maximum close to *p*/*p*^sat^ = 0.6. A different trend is however observed for the three isotherms at higher temperature for *p*/*p*^sat^ > 0.6. In particular, at *T* = 629.8 K and maximum *p*/*p*^sat^, the thermodynamic model predicts the speed of sound quite accurately, but it increasingly underestimates its value with increasing temperature, resulting in a deviation of 4.5% at *T* = 644.5 K. In other words, the model accurately captures the ideal gas departure at *T* = 629.8 K and *p*/*p*^sat^ = 0.9, but it overestimate all the values of ideal gas departure along the *T* = 644.5 K isotherm, leading to an underestimation of the speed of sound. This observation is relevant with respect to the possibility that D_6_ is a BZT fluid because these thermodynamic states at high reduced temperature and pressure close to saturation correspond to states for which the thermodynamic model predicts that the value of the fundamental derivative of gas dynamics Γ becomes negative. However, these measurements were performed at constant temperature, and Γ depends on the variation of speed of sound at constant entropy, therefore no information can be drawn from these measurements alone.

## 5 Conclusions

Measuring thermodynamic properties of complex organic molecules in the dense vapor phase is very challenging because measurements must be performed at high temperature and close to the thermal stability limit of these molecules in contact with suitable containing materials. This possibly explains why data of this type are extremely rare and none is known to the authors for organic molecules with a high degree of complexity. Among the property measurements that are most useful for the development of thermodynamic property models of fluids, sound speed stands out because it provides a direct relation to heat capacities, which are difficult to measure directly. If measured for dense vapor thermodynamics states, the speed of sound provides information also on the departure of properties from those that are related by the ideal gas assumption.

Moreover, knowing the value of the speed of sound is fundamentally important in gas dynamics in general and, for states whose relations among properties depart significantly from those of the ideal gas, in nonideal compressible fluid dynamics (NICFD), a relatively novel branch of fluid mechanics. Siloxane D_6_, being a sufficiently complex organic molecule is predicted to exhibit so-called nonclassical gasdynamic behavior in the dense vapor phase, and this is the main reason that motivated this experimental study.

The outcome of the investigation documented in this article can be summarized as follows:

- The possibility of measuring the speed of sound of fluids formed by complex organic molecules at dense vapor states has been demonstrated. For this purpose, a new prismatic resonator has been designed, realized, and successfully commissioned.
- The first measurement campaign has been carried out. The speed of sound and, in addition, the density of siloxane D_6_ have been measured along eight isotherms (temperatures between 555 and 645 K) in the dense vapor phase for states with 0.44 < *Z* < 0.95 and starting from states very close to saturation. The relative uncertainties of such measurements have been estimated to be 0.14% for the sound speed and between 0.2 kg·m^–3^ and 1.2 kg·m^–3^ for the density.
- The measured values have been compared with values calculated with the iPRSV cubic equation of state model as available at the time of the measurements in order to verify the consistency of the experimental data and highlight the limitations of currently available models. It was thus shown that the model correctly predicts the density given the temperature and the pressure for states characterized by a pressure ratio *p*/*p*^sat^ up to 0.7. However, the model underestimates the density by as much as 5% for *p*/*p*^sat^ ≈ 0.9. iPRSV speed of sound predictions however all lie within −2% and 0% of the measured value, except for the states along the isotherm at the highest temperature (645 K), for which model predictions differ from measured values by as much as −4%; this deviation may be reduced by performing critical point and dew line measurements at high reduced temperature *T*_r_.
- The measurement accuracy can be improved with some modifications to the resonator body. First, the shape of the cavity should be modified to be a circular cross-section. This change could be achieved without significantly modifying the current resonator for instance by adding a circular tube which has the length of the cavity and the diameter of the acoustic excitation system. This improvement is three-fold: the acoustic actuator shape would match that of the cavity, thus stronger acoustic waves would be generated; the volume-to-area ratio would be more favorable, thus boundary layer losses would be weaker; the dead volume between the square cavity and the tube could be filled with a fluid such as pressurized air, or even liquid D_6_, to serve as a thermal bath and enhance the temperature homogeneity inside the cavity. A second modification would be the replacement of the current fluid injection port by a smaller capillary tube to reduce the uncertainty on the estimation of the density which is a function of the unknown amount of liquid fluid contained in the inlet pipe. Finally, a solution should be elaborated to ensure the complete bleeding of the various pipes, which was not found to be possible with the current setup.
